# In the heart of the city: *Trypanosoma cruzi* infection prevalence in rodents across New Orleans

**DOI:** 10.1186/s13071-020-04446-y

**Published:** 2020-11-14

**Authors:** Bruno M. Ghersi, Anna C. Peterson, Nathaniel L. Gibson, Asha Dash, Ardem Elmayan, Hannah Schwartzenburg, Weihong Tu, Claudia Riegel, Claudia Herrera, Michael J. Blum

**Affiliations:** 1 Department of Ecology & Evolutionary Biology, University of Tennessee, Knoxville, TN USA; 2 Department of Tropical Medicine, Vector-Borne Infectious Disease Research Center, Tulane University, School of Public Health and Tropical Medicine, New Orleans, LA USA; 3 City of New Orleans Mosquito, Termite, Rodent Control Board, New Orleans, LA USA

**Keywords:** Abandonment, Chagas disease, Commensal pest, Hurricane Katrina, Mice, Public health, Rats, Urban zoonosis

## Abstract

**Background:**

*Trypanosoma cruzi* - the causative agent of Chagas disease - is known to circulate in commensal pests, but its occurrence in urban environments is not well understood. We addressed this deficit by determining the distribution and prevalence of *T. cruzi* infection in urban populations of commensal and wild rodents across New Orleans (Louisiana, USA). We assessed whether *T. cruzi* prevalence varies according to host species identity and species co-occurrences, and whether *T. cruzi* prevalence varies across mosaics of abandonment that shape urban rodent demography and assemblage structure in the city.

**Methods:**

Leveraging city-wide population and assemblage surveys, we tested 1428 rodents comprising 5 species (cotton rats, house mice, Norway rats, rice rats and roof rats) captured at 98 trapping sites in 11 study areas across New Orleans including nine residential neighborhoods and a natural area in Orleans Parish and a neighborhood in St. Bernard Parish. We also assayed Norway rats at one site in Baton Rouge (Louisiana, USA). We used chi-square tests to determine whether infection prevalence differed among host species, among study areas, and among trapping sites according to the number of host species present. We used generalized linear mixed models to identify predictors of *T. cruzi* infection for all rodents and each host species, respectively.

**Results:**

We detected *T. cruzi* in all host species in all study areas in New Orleans, but not in Baton Rouge. Though overall infection prevalence was 11%, it varied by study area and trapping site. There was no difference in prevalence by species, but roof rats exhibited the broadest geographical distribution of infection across the city. Infected rodents were trapped in densely populated neighborhoods like the French Quarter. Infection prevalence seasonally varied with abandonment, increasing with greater abandonment during the summer and declining with greater abandonment during the winter.

**Conclusions:**

Our findings illustrate that *T. cruzi* can be widespread in urban landscapes, suggesting that transmission and disease risk is greater than is currently recognized. Our findings also suggest that there is disproportionate risk of transmission in historically underserved communities, which could reinforce long-standing socioecological disparities in New Orleans and elsewhere.
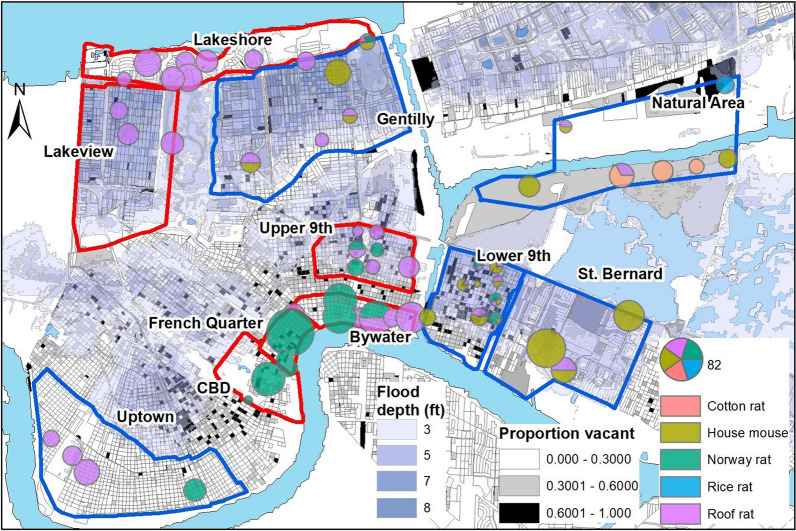

## Background

Chagas disease, caused by the zoonotic protozoan *Trypanosoma cruzi*, is arguably the most prevalent and widespread parasitic disease in the Western Hemisphere [1]. Most of the disease burden occurs in Central and South America, where there are more than 8 million current infections and more than 30 thousand new infections reported per year [2, 3]. There is growing concern that the disease will become prevalent in the USA with shifting climate conditions and continuing land use intensification [4]. Though only 31 autochthonous cases have so far been reported in the USA [5–8], wildlife surveillance has detected *T. cruzi* in over two dozen mammal species, and at least 11 species of triatomine vectors have been recorded from 29 states [9], raising the possibility that *T. cruzi* is already endemic and widespread [10–14].

While the occurrence of all autochthonous cases of *T. cruzi* infection in rural and semi-rural areas of the USA has sustained long-standing interest in transmission risk associated with sylvatic and peri-urban ecotonal habitats, there is growing evidence of transmission risk within densely populated cities. A number of hosts, for example, are commensal pests that frequently occur in urban areas [2]. Some triatomine vectors carrying *T. cruzi* have been detected in major city centers [15–19], and others are known to have become adapted to urban habitats [10, 14]. Evidence of interactive connectivity between reservoirs and vectors [20] has reinforced concerns that *T. cruzi* can readily invade cities from peri-urban or peripheral natural areas [21, 22]. Reports of infected commensal and peri-domestic mammals (e.g. opossums, raccoons and rodents) in urban areas of the Caracas Valley of Venezuela and southeastern Louisiana (e.g. shelter dogs, raccoons and rodents) support the supposition that Chagas disease is an emergent urban zoonosis [23–26], highlighting the need for further understanding of infection prevalence in hosts that reside within USA cities [20, 26].

Rodents are among the most widespread and abundant pathogen reservoirs in cities [27], and thus often serve as indicators of transmission risk across urban landscapes. Though it is well understood that commensal species like Norway rats (*Rattus norvegicus*), roof rats (*Rattus rattus*) and house mice (*Mus musculus*) can carry *T. cruzi*, remarkably little is known about infection prevalence in urban populations, particularly within the USA [13, 14]. So far, there have been more extensive studies of other sentinel hosts commonly found in cities, such as shelter dogs and raccoons [28–30]. Smaller scale assays of rodents conducted in South America (e.g. Brazil, Venezuela) have illustrated that *T. cruzi* infection prevalence can be quite high in some commensal species, including Norway rats [25, 31]. Likewise, a recent assay of rodents from southeastern Louisiana detected *T. cruzi* in house mice from an urban site in New Orleans [32], raising the possibility that it might occur elsewhere in the city.

Here we report findings from a geographically extensive survey of *T. cruzi* infection in urban and peri-urban populations of rodents captured from New Orleans, with reference to a site in Baton Rouge (Louisiana, USA). Our aim was to address long-standing concerns about transmission risk in the area; one of the first autochthonous cases of Chagas disease in the US occurred near New Orleans [33], and surveys of rodents in proximate sylvatic and rural areas have found evidence of high levels of *T. cruzi* infection prevalence (e.g. 76%) [14]. Recent surveys also have found evidence that other rodent-borne pathogens of human concern (e.g. *Angiostrongylus cantonensis*, *Bartonella spp*., *Leptospira spp*.) are widespread throughout the city [34–36], and that pathogen transmission risk has been shaped by discriminatory public policies executed in the wake of Hurricane Katrina that have reinforced long-standing legacies of socioecological disparities [37–39]. Accordingly, to better understand *T. cruzi* transmission risk, we assessed (i) infection prevalence in urban and peri-urban rodent populations overall as well as by species and location; and (ii) whether *T. cruzi* prevalence varies according to host attributes, species identity and species co-occurrence; and (iii) whether *T. cruzi* prevalence varies across mosaics of abandonment that shape urban rodent demography and assemblage structure in the city.

## Methods

### Study area and rodent trapping

**Fig. 1:**
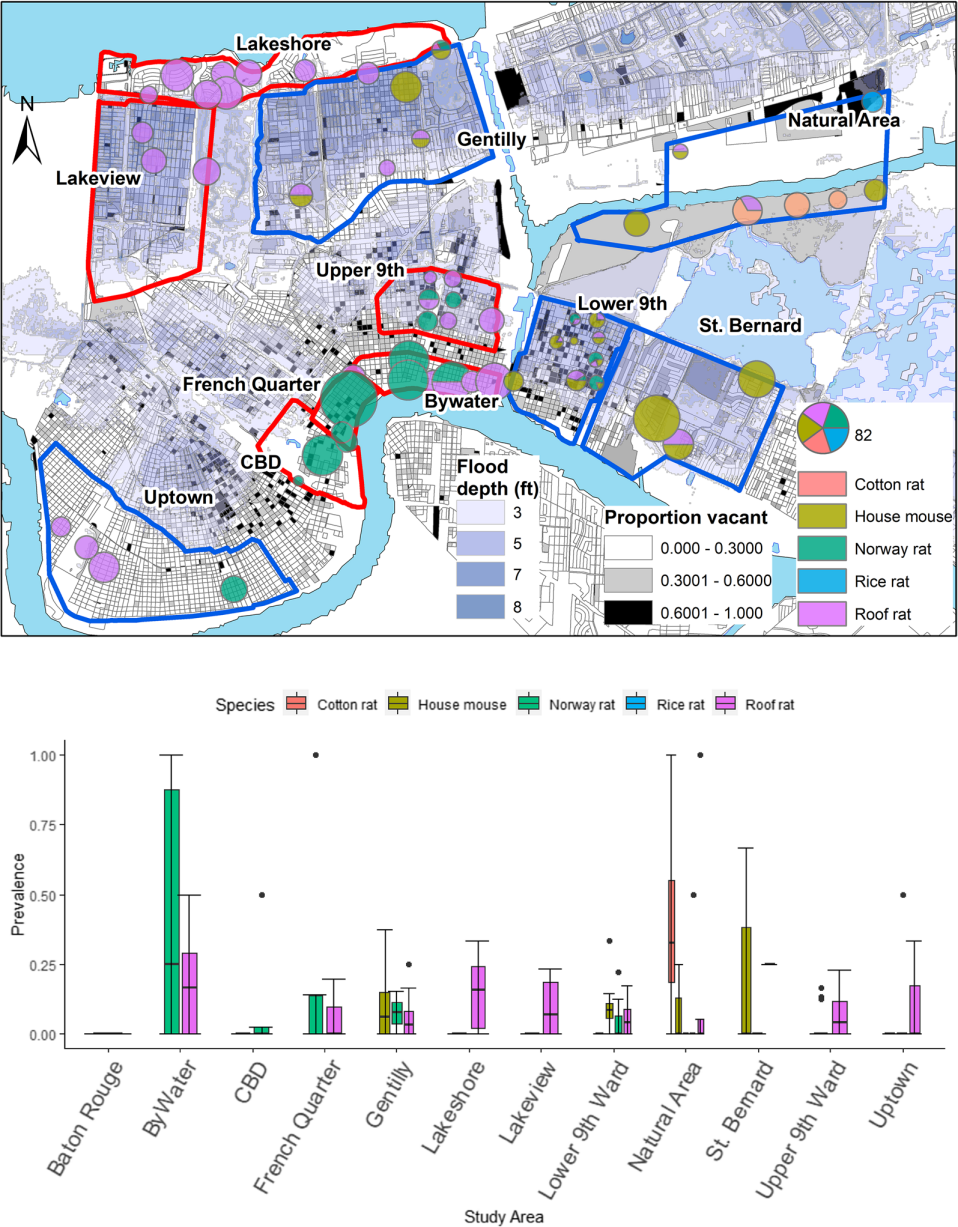
(Top) Map of *T. cruzi* infection prevalence by rodent species across Orleans Parish and St. Bernard Parish, with Tomahawk-only trapping areas outlined in red and Tomahawk + Sherman trapping areas outlined in blue (Bottom) *T. cruzi* infection prevalence in each host species by study area- colors indicate species, the middle horizontal line represents the median, bars represent the first and third quartiles, vertical lines represent the minimum and maximum range relative to the quartiles, and dots are outliers. *Abbreviation*: CBD, Central Business District

As described in detail by Peterson et al. [38], we conducted quantitative population and assemblage surveys of rodents from May 2014 to February 2017 following Tulane University IACUC protocols #0451 and #0460. We systematically trapped for larger rodents (i.e. rats) at 96 sites in 10 study areas, including eight neighborhoods in New Orleans, an adjacent natural area in Orleans Parish and an adjacent neighborhood in St. Bernard Parish (Table 1, Fig. 1) [34, 38]. Trapping sites corresponded to city blocks or the equivalent [34, 36, 38]. The study areas were selected to capture contrasting levels of income, flooding, and post-disaster landscape management after Hurricane Katrina in 2005 [34, 38]. During each trapping bout, 30 Tomahawk live traps (Tomahawk Live Trap, Hazelhurst, WI, USA) were set per site [38]. At a subset of 48 sites (Table 1, Fig. 1), an equal number of Sherman traps (H.B. Sherman Traps, Inc., Tallahassee, FL, USA) were set to capture smaller rodents (i.e. mice) [38]. To enable further comparisons, 13 additional sites were opportunistically sampled in coordination with the City of New Orleans Mosquito, Termite, and Rodent Control Board, including six sites in the Central Business District (CBD) and a Norway rat colony in Baton Rouge. For this study, we included rodents from 99 of the 109 trapping sites (Table 1). We measured standard weight and length of all trapped individuals, as well as species identity, sex, sexual maturity, and parity in females [38]. We categorized all Norway rats and roof rats into 3 age classes (juvenile, subadult and adult) according to body weight [40, 41]. Blood, urine, lung, liver, spleen, kidney, and tail tissues were sampled and archived in – 80 °C freezers.

**Table 1:** Prevalence of *T. cruzi* infection (number positive/number tested, followed by % in parentheses) in rodents by species and across all species sampled in New Orleans and Baton Rouge (Louisiana), with reference to the total number of sites where rodents were trapped and assayed for *T. cruzi* in each study area, followed (in parentheses) by the number of sites in each study area where Sherman trapping was conducted

Study area	No. of sites	Norway rat	Roof rat	House mouse	Cotton rat	Rice rat	Total
Bywater	9 (0)	3/9 (33)	6/26 (23)	–	–	–	9/35 (26)
CBD	5 (0)	3/44 (7)	–	0/1 (0)	–	–	3/45 (7)
French Quarter	6 (0)	7/52 (13)	1/8 (13)	–	–	–	8/60 (13)
Gentilly	10 (10)	2/14 (14)	6/89 (7)	8/55 (15)	–	–	16/158 (10)
Lakeshore	10 (0)	0/1 (0)	10/71 (14)	–	–	–	10/72 (14)
Lakeview	7 (0)	0/1 (0)	16/77 (21)	–	–	–	16/78 (21)
Lower 9th Ward	10 (10)	10/159 (6)	9/177 (5)	21/239 (9)	0/4 (0)	–	40/579 (7)
Natural area	8 (8)	0/2 (0)	2/22 (9)	4/43 (9)	5/16 (31)	1/7 (14)	12/90 (13)
St. Bernard Parish	7 (7)	0/1 (0)	1/4 (25)	21/52 (40)	–	–	22/57 (39)
Upper 9th Ward	16 (0)	5/78 (6)	8/98 (8)	–	–	–	13/176 (7)
Uptown	10 (10)	2/13 (15)	7/45 (16)	0/4 (0)	–	–	9/72 (13)
Baton Rouge	1 (0)	0/6 (0)	–	–	–	–	0/6 (0)
Total	99 (44)	32/380 (8)	66/627 (11)	54/394 (14)	5/20 (25)	1/7 (14)	158/1428 (11)

*Abbreviation*: CBD, Central Business District

### Site characterization

As described by Peterson et al. [38], flooding history, land use and socio-demography were characterized for each trapping site in the New Orleans metro area. Surface elevation, a proxy for flooding depth, was estimated from LiDAR with reference to water level data from Lake Pontchartrain [42]. We used Google Earth to estimate the proportion of vacant lots (i.e. vacancy) at each site. We also conducted plot-based assays of abandonment to document unmaintained vegetation, unmaintained buildings, and debris piles at each site. Additionally, land cover classification of tree cover, open grass, urban surface/bare soil, buildings, and open water was performed on three multispectral 0.5 m resolution satellite images taken in mid-to-late March 2014, 2015 and 2016. We used 2010 US Census data to derive information on block-level human population size and household income.

### Infection assay

Infection prevalence was determined according to a diagnostic PCR-based assay [43]. Following the manufacturer’s protocol, we used Qiagen blood and tissue kits to extract genomic DNA from blood samples premixed with the same volume of guanidine HCL 6M and EDTA 0.2M (pH 8). DNA was also extracted from 4 different laboratory-maintained reference strains of *T. cruzi*, for use as positive controls. All DNA extractions were tested for the presence of *T. cruzi* through PCR amplification of highly repetitive nuclear satellite DNA (satDNA) using primers TcZ1 and TcZ2 [43]. Infection status was determined based on electrophoresis of PCR products on an ethidium bromide stained 2.0% agarose gel run for 50 min at 100 V, with the presence of a 188-bp band qualifying a sample as positive for *T. cruzi* [26], confirmed through comparisons to negative controls.

### Statistical analysis

We characterized the prevalence of *T. cruzi* infection for all trapped species to report occurrence and distribution across all study sites, but all subsequent statistical analyses focused on Norway rats, roof rats and house mice due to low sample sizes for other species. We conducted all statistical analyses using R version 3.6.0.

As done in a similarly minded study of rat lungworm in rodents trapped across New Orleans [34], we used a chi-square approach to test for potential differences in *T. cruzi* infection prevalence. Pairwise tests were run to compare prevalence according to host species, with the level of significance Bonferroni corrected to account for multiple comparisons. Likewise, we used pairwise chi-square tests to determine whether infection prevalence differed among study areas and among sites categorized according to the number of host species present, with the level of significance Bonferroni corrected to account for multiple comparisons.

Following a prior study of rodent assemblage structure across New Orleans [38], we used generalized linear mixed models (glmm) with a binomial distribution to assess individual level and socioecological predictors of *T. cruzi* infection for all rodents and each host species, respectively. We used a set of predictors that have been found to be informative in past studies of rodents in New Orleans [38]. The global model of infection status included year, season, species (for the model describing infection in all rodents), sex, flooding depth, median household income, number of residents per block (or equivalent), remotely-sensed estimates of tree cover and the proportion of vacancy at each study site, as well as survey-based estimates of unmaintained buildings, debris, unmaintained vegetation, and the interactions of household income × flooding depth, unmaintained buildings × flooding depth, vacancy × flooding depth, vacancy × season, and vacancy × unmaintained vegetation as fixed effects. We included site as a random effect to account for repeated measurement of rodent abundance at each site over the study period [38]. For each analysis, all possible models were generated and ranked according to AIC score using the package *MuMin* in R. The top-selected model was determined according to the lowest AIC score.

## Results

### Prevalence of *T. cruzi* infection by species and location

We found that 158 (11%) of 1428 rodents tested positive for *T. cruzi*. The protozoan was detected in all host species; of the 627 roof rats tested, 66 (11%) were positive for *T. cruzi*, whereas 32 (8%) of 380 Norway rats, 54 (14%) of 394 house mice, 5 (25%) of 20 cotton rats (*Sigmodon hispidus*), and 1 (14%) of 7 rice rats (*Oryzomys palustris*) tested positive. Infection prevalence was not significantly different between species (*χ*^2^ = 9.7, *P* > 0.23). Infected individuals were found in all of the Orleans and St. Bernard Parish study areas, but not at the Baton Rouge site (Fig. 1; Additional file 1: Figure S1; Table 1). Overall infection prevalence (38.6%) was significantly higher in St. Bernard Parish than in the CBD (*P* < 0.03), Gentilly (*P* < 0.001), Lower 9th Ward (*P* < 0.001) and Upper 9th Ward (*P* < 0.001) neighborhoods in Orleans Parish (Fig. 1). Overall prevalence also was higher in the ByWater than in the Lower 9th Ward neighborhood (*P* < 0.02) (Fig. 1). Prevalence in the natural area did not differ from levels observed in other study areas (Table 1) (all, *P* > 0.05). Prevalence of infection in Norway rats did not differ among the study areas (*χ*^2^ = 12.9, *P* > 0.29), whereas roof rats exhibited higher prevalence in the Lakeview neighborhood compared to the Lower 9th Ward (*χ*^2^ = 22.65, *P* < 0.02) (Fig. 1, Table 1). House mice exhibited higher infection prevalence in the St. Bernard Parish neighborhood compared to individuals captured in the Lower 9th Ward (*P* < 0.001) and the natural area (*P* < 0.02) (Fig. 1, Table 1).

### Environmental and individual predictors of infection

Individual level characteristics were not especially informative predictors of infection. Infection status did not differ according to sex overall (*χ*^2^ = 0.94, *P* = 0.33) or by species (Norway rat: *χ*^2^ = 1.35, *P* = 0.25; roof rat: *χ*^2^ = 0.11, *P* = 0.74; house mouse: *χ*^2^ = 0.07, *P* = 0.79). Infection status did not vary by body mass for roof rats or house mice, although smaller Norway rats were more likely to be infected (*t* = 2.03, *P* = 0.049). Infection also did not differ by age group for Norway rats or roof rats (*χ*^2^ = 3.10, *P* = 0.21; *χ*^2^ = 1.99, *P* = 0.37, respectively).

Total infection prevalence varied according to the presence of different host species at trapping sites. Considering sites with both Sherman and Tomahawk traps (Fig. 1), average prevalence of *T. cruzi* was higher (*χ*^2^ = 18.23, *P* = 0.001) at sites with 2 species (22%) than sites with 3 species (9%) (Additional file 1: Figure S2). When looking at different combinations of species at a site, there was higher (*χ*^2^ = 9.93, *P* = 0.001) average prevalence at sites with Norway rats and house mice (42%) than sites with roof rats and house mice (14%). Notably, no relationship was found between total host abundance and infection prevalence at a site. When evaluating data from just Tomahawk traps, we found that average prevalence of *T. cruzi* was similar (*χ*^2^ = 33.73, *P* = 0.21) at sites with 1 species (10%) and with 2 species of rat (15%), and there was no relationship between the number of animals trapped and infection prevalence.

**Fig. 2:**
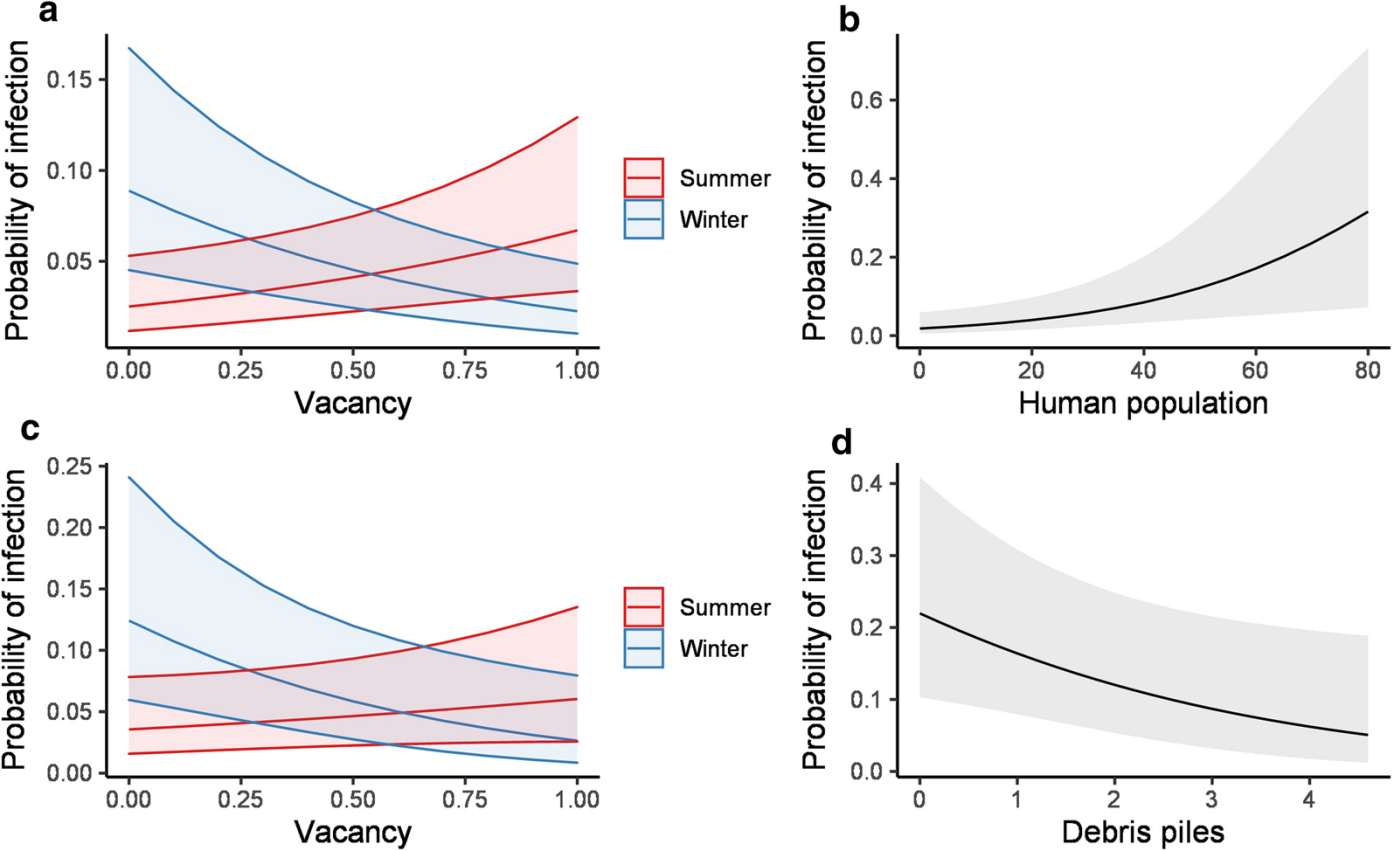
Probability of infection of (**a**) all rodents (**b**) Norway rats (**c**) roof rats and (**d**) house mice according to socioecological predictors reflecting measurements taken for each trapping site. Shaded areas correspond to 95% confidence intervals

The top selected model of overall infection status was > 2 ΔAIC than the next best model, and thus it is the only model for which results are presented [38]. For all species, year of sampling (coef.  = 0.97, *P* = 0.001), vacancy (coef.  = 1.02, *P* = 0.01), season (coef.  = 1.33, *P* < 0.001), flood depth (coef.  = − 0.12, *P* < 0.05), and the interaction of season × vacancy (coef.  = − 2.47, *P* < 0.001) were significant predictors of infection. The model indicates that animals trapped during winter in areas with higher vacancy were 2 times less likely to test positive for *T. cruzi*, whereas animals trapped during summer in areas with higher vacancy were 2 times more likely to test positive (Fig. 2a). When looking at each species independently, human population (coef.  = 0.04, *P* < 0.01), unmaintained vegetation (coef.  = − 4.19, *P* < 0.01) and year (coef.  = 1.37, *P* < 0.02) were predictors of infection in Norway rats (Fig. 2b); season (coef.  = 1.34, *P* < 0.001), unmaintained vegetation (coef.  = − 0.86, *P* < 0.05), year (coef.  = 0.99, *P* < 0.01) and the interaction of vacancy × season (coef.  = − 2.1, *P* < 0.01) were predictors of infection in roof rats (Fig. 2c); whereas debris (coef. = − 0.36, *P* < 0.05) was the only significant predictor of infection in house mice (Fig. 2d). Overall infection prevalence was significantly lower during 2014 than during 2015 and 2016, which reflects variation in Norway and roof rats, as mice were only sampled during 2015 and 2016.

## Discussion

Here we present the most comprehensive report, thus far, of *T. cruzi* infection prevalence across an urban landscape, providing further support for the supposition that Chagas disease is an emerging urban zoonosis [23, 44, 45]. Despite evidence of transmission risk within densely populated cities elsewhere [23–26], work describing the prevalence of *T. cruzi* in the USA has largely focused on natural and peri-urban areas [13] like those where autochthonous cases of Chagas disease have been detected [46]. Our results demonstrate that *T. cruzi* is circulating in urban rodent populations across New Orleans at an overall prevalence of 11%. We detected *T. cruzi* in all host species in all study areas in New Orleans, including densely populated tourist areas like the French Quarter. Whereas infection prevalence did not differ by host species, it did vary by study area and trapping site, reflecting variation in prevalence within species and differences in the co-occurrence of host species, which echoes broader patterns of rodent assemblage structure across the city [38]. Prevalence also appears to be mediated by features of disaster-driven counter-urbanization in the study area [34, 38, 39]. Evidence that *T. cruzi* can be widespread and abundant in urban landscapes suggests that transmission risk in the USA is greater than is currently recognized. Our findings also indicate that it is likely there is disproportionate risk of transmission in historically underserved communities, which could further reinforce long-standing legacies of socioecological disparities in New Orleans and elsewhere [37, 38, 47].

There are some notable similarities in the epidemiology of *T. cruzi* infection in New Orleans and urban areas where the *T. cruzi* is considered to be endemic. For example, we detected *T. cruzi* in all five of the rodent host species found in New Orleans [39], which parallels reports of *T. cruzi* infections in multiple rodent and other mammalian host species trapped in residential neighborhoods in the Yucatan [48] and Caracas Valley [24, 25]. We also found a comparable level of overall infection prevalence in New Orleans [25, 48, 49]. It is notable, however, that we did not find differences in prevalence among host species. Greater variation has been found elsewhere, including among urban populations of commensal rodents. For example, surveys in the Yucatan and Caracas Valley found that *T. cruzi* was more prevalent in roof rats than in house mice [25, 48]. Likewise, no evidence of infection was found in rodents trapped in forest fragments in Salvador (Brazil), where only *Didelphis* opossums were found to carry *T. cruzi* [49]. More extensive surveys of urban hosts in endemic areas might reveal greater parity in infection prevalence; however, as most prior work has involved comparisons among a much smaller number of trapped individuals than the number of rodents assayed for this study [24, 25, 48–50]. On the other hand, more extensive surveys targeting less abundant species native to New Orleans (i.e. cotton rats and rice rats) might reveal evidence of variation in infection prevalence among hosts.

Detection of infected rodents in all of our study areas indicates that transmission among zoonotic hosts is local and widespread across New Orleans. Though the possibility of local transmission has been raised by prior work on other urban commensal and domestic mammal species [23], it has been difficult to exclude alternatives like geographical transience and interactive connectivity between reservoirs and vectors [20] located in peripheral habitats [49]. In part, this is because limited trapping as well as a focus on hosts with large range sizes and high mobility has prevented identification of the point(s) of infection. This is well illustrated by a recent study [26] conducted in New Orleans and Baton Rouge that found evidence of high infection prevalence (23–43%) in raccoons (*Procyon lotor*), which can readily travel between urban and nearby natural areas [51]. Commensal rodents, on the other hand, typically move < 200 m from their nest [52–55]. Accordingly, infected individuals must have acquired *T. cruzi* close to the respective trapping location, indicating that at least one vector is also likely in the area. Surveys have not yet been conducted to assay the incidence of known vectors in New Orleans, but the possibility of local transmission is supported by surveys that have detected the vector *Triatoma sanguisuga* in urban areas in Texas and Tennessee [56, 57]. Prior work elsewhere also indicates that other vectors (e.g. *Triatoma infestans*) can migrate from rural environments and subsequently become endemic to urban areas [58]. Determining the occurrence of vectors in New Orleans would offer a more complete characterization of local transmission cycle(s) and thus clarify the risk of transmission to humans across the city. Likewise, further assessment of conditions in Baton Rouge would help resolve apparent inconsistencies between our results and evidence from prior studies showing that racoons harbor high levels of infection across the city [26].

Our findings indicate that biotic and landscape features shape infection prevalence in rodent hosts across the New Orleans metro area. With little exception, the likelihood of infection did not differ according to individual level host attributes. We nonetheless found evidence indicating that infection varied within some species and that prevalence differed according to host co-occurrence, dependent on the particular host species present. This result highlights the need for further understanding of how host co-occurrence and abundance (i.e. the factors governing assemblage structure) influences *T. cruzi* transmission in cities. Prevalence also appears to be mediated by disaster-driven counter-urbanization that has progressed in the study areas since Hurricane Katrina in 2005 [35, 39, 59]. Catastrophic flooding, discriminatory resettlement and rebuilding policies, and municipal differences in land management have given rise to a socioecological mosaic of abandonment across New Orleans [37, 38]. Overall and individual species-level prevalence strongly reflected elements of abandonment including the severity of flooding disturbance (i.e. depth), vacancy, and debris at trapping sites. The same factors also appear to shape rodent demography and assemblage structure in the city [38, 39], raising the possibility that the recovered relationships with *T. cruzi* infection prevalence are simply a secondary phenomenon (i.e. a byproduct of variation in rodent demography and assemblage structure). However, we found that the influence of counter-urbanization (i.e. vacancy) varies by season (Fig. 2), which is consistent with indications from surveys of other mammalian hosts in New Orleans [26] that infection prevalence is seasonally dynamic. This illustrates that the likelihood of infection is not just a function of rodent host responses to habitat or resource availability. Rather, it also probably reflects habitat-mediated variation in vector occurrence [17, 18] or interactions between hosts, vectors, and *T. cruzi* parasites [60]. Further work is thus warranted to better understand how socioecological factors shape the urban epidemiology of *T. cruzi*.

Disparities in disaster-driven abandonment across New Orleans [37, 38, 47] indicate that there is likely greater risk of pathogen transmission in historically underserved communities [34]. Our findings suggest, however, that transmission risk is highly heterogeneous within and among neighborhoods due to variation in site-level characteristics that govern infection prevalence. This is well illustrated by comparisons of the Lower 9th Ward to adjacent areas in St. Bernard Parish (Fig. 1). Both neighborhoods are burdened by comparably high levels of disaster-driven vacancy but exhibit contrasting habitat characteristics and rodent assemblages, reflecting differences in post-Katrina municipal land management [37, 38]. The overall prevalence of *T. cruzi* infection also differs between the neighborhoods (Fig. 1, Table 1), indicating that transmission risk is not simply a function of vacancy, but that it also depends on local habitat conditions (e.g. maintained *versus* unmaintained vegetation, the occurrence of favored plant species, etc.) that may govern tritrophic interactions. Further study that reveals the scale of associations would better define transmission risk and shed light on whether proactive measures (e.g. rodent control, removing debris and maintaining vegetation) can be taken to reduce infection prevalence [61]. Likewise, understanding variation in infection intensity and the diversity of *T. cruzi* carried by vectors and hosts [14, 32] could help alleviate potential risks to human well-being in cities across the USA and elsewhere [2, 23, 62].

## Conclusions

Our study provides further support for prior claims that Chagas disease is an emerging urban zoonosis. Our results illustrate that *T. cruzi* is surprisingly widespread in New Orleans, suggesting that transmission risk in the USA and elsewhere is greater than is currently recognized. Evidence of seasonal dynamics in areas burdened by abandonment also indicates that there is disproportionate risk of transmission in historically underserved communities, which could reinforce long-standing socioecological disparities in the USA and elsewhere [37, 38, 47].

## Supplementary information

**Additional file 1: Figure S1.** Infection prevalence across all rodent species by study area with individual sites noted as jitter dots. The middle horizontal line represents the median, bars represent the first and third quartiles, vertical lines represent the minimum and maximum range relative to the quartiles, and dots are outliers. *Abbreviation*: CBD, Central Business District. **Figure S2.** Variation in *T. cruzi* infection prevalence according to the number of host species at Tomahawk + Sherman trapping sites. The middle horizontal line represents the median, bars represent the first and third quartiles, vertical lines represent the minimum and maximum range relative to the quartiles, and dots are outliers.

## Data Availability

Data generated specifically for this study are included within the article and its additional file. Data generated on individual attributes, rodent assemblage structure, as well as site-level demography, land use and landscape features that were utilized in analyses presented in this study are available online or in related publications [34, 38]. Those that are not publicly available are available from the corresponding author upon reasonable request.

## Abbreviations

AIC: Akaike information criteria
CBD: Central business district
DNA: Deoxyribonucleic acid
EDTA: Ethylenediaminetetraacetic acid
FL: Florida
GLMM: Generalized linear mixed model
HCL: Hydrochloric
IACUC: Institutional Animal Care and Use Committee
PCR: Polymerase chain reaction
satDNA: Nuclear microsatellite DNA
WI: Wisconsin
