# Supplementary material for: In the heart of the city: Trypanosoma cruzi infection prevalence in rodents across New Orleans
Source: Parasit Vectors. 2020 Nov 14;13:577. doi: 10.1186/s13071-020-04446-y (PMC7666460; doi:10.1186/s13071-020-04446-y)
Supplement: Supplementary file 1 — Additional file 1: Figure S1. Infection prevalence across all rodent species by study area with individual sites noted as jitter dots. The middle horizontal line represents the median, bars represent the first and third quartiles, vertical lines represent the minimum and maximum range relative to the quartiles, and dots are outliers. Abbreviation: CBD, Central Business District. Figure S2. Variation in T. cruzi infection prevalence according to the number of host species at Tomahawk + Sherman trapping sites. The middle horizontal line represents the median, bars represent the first and third quartiles, vertical lines represent the minimum and maximum range relative to the quartiles, and dots are outliers. [file 13071_2020_4446_MOESM1_ESM.docx]

**Additional file 1**

**Figure S1.** Infection prevalence across all rodent species by study area with individual sites noted as jitter dots. CBD = Central Business District. The middle horizontal line represents the median, bars represent the first and third quartiles, vertical lines represent the minimum and maximum range relative to the quartiles, and dots are outliers.

**Figure S2.** Variation in *T. cruzi* infection prevalence according to the number of host species at Tomahawk + Sherman trapping sites. The middle horizontal line represents the median, bars represent the first and third quartiles, vertical lines represent the minimum and maximum range relative to the quartiles, and dots are outliers.
